# Randomized Cross‐Over Analysis of the Influence of Nitrogen Multiple Breath Washout on Spirometry in Monitoring Lung Function in Patients With Cystic Fibrosis and Primary Ciliary Dyskinesia

**DOI:** 10.1002/ppul.71189

**Published:** 2025-07-10

**Authors:** Anna Charlotte Schoop, Robin Denz, Christoph Maier, Folke Brinkmann, Thomas Lücke, Anne Schlegtendal

**Affiliations:** ^1^ Department of Paediatric Pneumology University Children's Hospital, Katholisches Klinikum Bochum, Ruhr‐University Bochum Bochum Germany; ^2^ Department of Medical Informatics, Biometry and Epidemiology Ruhr‐University Bochum Bochum Germany; ^3^ University Children's Hospital, Katholisches Klinikum Bochum, Ruhr‐University Bochum Bochum Germany; ^4^ University Medical Center Schleswig‐Holstein, Campus Centrum Lübeck, The University of Lübeck Lübeck Germany; ^5^ Airway Research Center North (ARCN) of the German Center of Lung Research (DZL) Grosshansdorf Germany

**Keywords:** cystic fibrosis, multiple breath washout, primary ciliary dyskinesia, spirometry

## Abstract

**Background:**

When monitoring lung function in patients with Cystic Fibrosis (pwCF) and Primary Ciliary Dyskinesia (pwPCD), nitrogen multiple breath washout (N_2_MBW) is usually performed before spirometry to prevent forced expiratory maneuvers from altering N_2_MBW results. The N_2_MBW may affect spirometry if cooperation decreases after a long period of examination or due to prolonged oxygen inhalation. The equivalence of these concepts has never been investigated in a randomized cross‐over trial. We hypothesized that the order of pulmonary function tests (PFTs) would not influence the *z*‐score FEV_1_.

**Methods:**

A total of 47 clinically stable outpatients (36 pwCF, 11 pwPCD; 16.7 ± 8.1 years) were randomized into two groups. Each patient underwent N_2_MBW and spirometry at two consecutive visits (median interval 104 days): Group I: Spirometry followed by N_2_MBW (A), reversed order at the second visit (B), Group II reversed (B→A).

**Statistics:**

For the equivalence test, a change in *z*‐score FEV1 (primary endpoint) ±0.2 and lung clearance index (LCI_2.5_, secondary endpoint) ±15% was not considered relevant; therefore, changes outside this range were considered an intervention effect in the linear mixed model (*p* < 0.05).

**Results:**

There was a significant deterioration in *z*‐score FEV1 between the two appointments (period effect: −0.177; *p* = 0.012). The intervention effect and 95% confidence interval were within the equivalence range in both groups (*z*‐score FEV1: 0.039; −0.0765 to 0.1539, LCI_2.5_: −0.082; −0.3691 to 0.2054).

**Discussion:**

In our cohort the order of PFTs has no influence on the results suggesting that a greater flexibility in practice is possible without the risk of falsifying results.

**Trial Registration:**

German Clinical Trials Register (No. DRKS00027473).

## 1 Introduction

Cystic fibrosis (CF) and primary ciliary dyskinesia (PCD) are two rare, chronic, recessive inherited diseases: CF is caused by mutations in the gene encoding the cystic fibrosis transmembrane conductance regulator (CFTR), leading to impaired ion transport via this chloride channel with subsequent increased viscosity of the secretions of various surface epithelia of exocrine tissue especially in the respiratory tract [1, 2, 3]. PCD occurs due to various mutations causing heterogeneous structural and functional impairment of motile cilia [4, 5]. Although the pathophysiology is different, both diseases worsen mucociliary clearance, resulting in chronic upper and lower respiratory tract infections leading to progressive loss of lung function and the development of irreversible bronchiectasis [5, 6, 7, 8, 9].

Regular monitoring of lung function is important for clinical surveillance and detection of exacerbations to prevent progression of lung damage, such as bronchiectasis. Spirometry is currently the most widely used clinical monitoring tool, measuring central obstruction via the forced expiratory volume in 1 s (FEV_1_). However, various studies have reported that nitrogen multiple breath washout (N_2_MBW), measuring ventilation inhomogeneity via lung clearance index (LCI_2.5_), is a more sensitive method for detecting lung disease in patients with CF (pwCF) and patients with PCD (pwPCD) [10, 11]. As a result, N_2_MBW is increasingly becoming part of clinical routines and is recommended in current guidelines [12, 13].

It is common clinical practice to begin with N_2_MBW and perform spirometry second, as forced expiratory maneuvers during spirometry may mobilize secretions and therefore affect the following measurements [14]. But recommendations to the order of testing are missing. This usual sequence of lung function tests can often lead to long waiting times for the patient. Especially, the long examination times of N_2_MBW may lead to decreased compliance with further testing. Additionally, it is possible that inversely N_2_MBW may also influence results of spirometry, for example, the effect of breathing 100% oxygen during N_2_MBW on spirometry results is unknown.

There are only two studies using a test–retest design analyzing the influence of spirometry on MBW outcomes. Au and colleagues detected no short‐term effect in a small cohort of 15 inpatient children with CF (6–18 years), when N_2_MBW was performed before and immediately after spirometry [14]. In contrast, Subbarao and colleagues reported a significant decrease in LCI in sedated infants (4–30 months) with lung disease. However, as MBW in infants is performed with a sulfur hexafluoride/helium gas mixture and FEV_1_ is measured with raised volume rapid thoracoabdominal compression (RVRTC), it is difficult to compare the procedures [15].

To the best of our knowledge, the influence of N_2_MBW and spirometry maneuvers on lung function results has not been measured in daily clinical practice in a randomized cross‐over study in relevant patient groups (pwCF and pwPCD).

Therefore, in children, adolescents, and adults with CF and PCD, we tested the hypothesis that measurement of lung function (LCI_2.5_) using N_2_MBW has no effect on FEV_1_ (*z*‐score) subsequently measured by spirometry. In addition, we analyzed as a secondary outcome whether the reverse order had a significant effect on the LCI_2.5_.

## 2 Methods and Materials

### 2.1 Study Design

This is a single‐center, prospective, randomized cross‐over study. Subjects were recruited at the CF and PCD outpatient clinic of the University Children′s Hospital of the Ruhr‐University Bochum, Katholisches Klinikum Bochum (Germany) from June 2021 to August 2022.

To minimize the burden on patients and increase their willingness to participate, the study visits were combined with regular follow‐up appointments at the PCD outpatient clinic. Parents, guardians, and patients were preinformed by telephone before their regular appointment and received further written and verbal information about the study at their first appointment. After approval, study patients were randomized into two groups and underwent spirometry and N_2_MBW one after the other according to their randomization. At the first visit, Group I performed spirometry first, followed by N_2_MBW (A). Group II started with N_2_MBW first, followed by spirometry (B). The order of measurements was reversed in both groups at the next consecutive visit (see below). If spirometry did not meet the American Thoracic Society (ATS) and European Respiratory Society (ERS) standard [16] and/or N_2_MBW did not agree with the international consensus statement by the *European Respiratory Journal* [17], patients were excluded from our secondary analysis (see below).

The study was approved by the Ethics Committee of the Ruhr‐University of Bochum (No. 21. 7210) on 03.28.2021 and registered at the “German Clinical Trials Register” (No. DRKS00027473). We used the CONSORT reporting guidelines [18].

### 2.2 Subjects

Inclusion criteria for pwPCD and pwCF were a confirmed diagnosis, age over 6 years, FEV_1_ over 60% predicted and informed consent from the subject. All pwCF had a genetically confirmed diagnosis. PCD diagnosis was verified by video microscopy, transmission electron microscopy, and immunofluorescence microscopy and/or genetics, according to ERS guidelines [19]. Patients were included if their diagnosis was classified as “PCD positive” according to the ERS guideline. Subjects were excluded if they were unwilling to participate, had insufficient German language skills or had not attended their appointments regularly over the last year. pwCF on new CFTR‐modulator therapy 6 weeks prior to enrollment and patients with an acute exacerbation 2 weeks prior to enrollment were enrolled at a later date. Acute exacerbation was defined according to the criteria by Fuchs et al. modified by the European consensus group [20] as the need for additional antibiotic treatment indicated by a recent change in at least two of the following: (1) change in sputum volume or color, (2) increased cough, (3) increased malaise, fatigue or lethargy, (4) anorexia or weight loss, (5) decrease in pulmonary function by 10% or more/radiographic changes, (6) increased dyspnea. Inclusion criteria were checked again at the second consultation and, if in doubt, follow‐up was postponed to the next appointment. All patients were on their standard medication with no changes between the two visits. Patient characteristics (including diagnostic criteria, pulmonary function level, and ethnicity) were collected from clinical records at baseline.

### 2.3 Randomization

Before recruitment, randomization lists were generated using R (version 4.2.1.), with block randomization using random block lengths of 2, 4, or 6 and stratification by mean *z*‐score FEV_1_ with a lower limit at *z* = −1 to optimize group homogeneity.

At their first appointment, patients were assigned to their group (I or II) in consecutive order according to the corresponding randomization list by A.C.S. Patients agreed to participate without knowing about the randomization.

### 2.4 Lung Function Tests

Each appointment was part of standard care examinations. These are carried out routinely every 3–6 months. In addition to lung function testing this includes measuring weight and height and measuring peripheral arterial oxygen saturation using a pulse sensor. Microbiological testing for respiratory pathogens was performed on sputum or throat swab from all patients. Spirometry was performed using MasterScreen Body, Vyaire, Hoechberg, Germany, according to ATS/ERS technical statement [16] and assessed and controlled by experienced, specially trained personnel [21, 22]. N_2_MBW was performed as nitrogen (N_2_) washout using the Exhalyzer D, EcoMedics AG, Duernten, Switzerland, according to the international consensus statement published by the *European Respiratory Journal* [17].

To analyze the influence of spirometry on LCI_2.5_, all N_2_MBW measurements underwent real‐time quality control by the N_2_MBW operator (A.C.S.) and were reevaluated by an experienced reviewer (A.S.). Patients whose lung function tests did not meet the quality criteria, for example, showed evidence of leak or irregular breathing pattern or similar [23], were excluded from this analysis.

Data were collected for all patients at both visits. For spirometry *z*‐scores for FEV_1_ and FVC were calculated according to the Global Lung Initiative reference values [24] and for N_2_MBW LCI_2.5_ was measured.

### 2.5 Statistics

Continuous variables were described using arithmetic means, standard deviations, ranges, and medians. Counts and percentages were used to describe categorical variables.

Bland–Altman plots were used to visualize possible systematic dependencies of the difference in mean values of *z*‐score FEV_1_ and LCI_2.5_ on the average scores.

The effect of N_2_MBW on spirometry (primary outcome, intervention effect = testing order N_2_MBW first, spirometry second) with *z*‐score FEV_1_ as the outcome variable was analyzed using a linear mixed model with measurement order and appointment number (first or second) as fixed effects and the patient as a random effect. To minimize sex, weight and age bias *z*‐score FEV_1_ was defined as the outcome variable [24]. The main hypothesis was formulated as an equivalence test, meaning that the mean and the upper and lower 95% confidence intervals must fall within the defined equivalence range. To define the equivalence range, we analyzed the mean change in *z*‐score FEV_1_ in 16 (10 pwCF, 6 pwPCD) randomly selected clinically stable example patients who had undergone regular spirometry measurements over a sufficient period of time. With a mean time interval of 91 days, the mean change in *z*‐score FEV_1_ for pwCF was −0.13 and for pwPCD with a mean time interval of 125 days, *z*‐score FEV_1_ increased by a mean of 0.22 points. Based on this preliminary analysis, the equivalence range was defined as ±0.2, meaning that an average change in *z*‐score FEV_1_ of < 0.2 is deemed to be negligible.

The influence of spirometry on N_2_MBW (secondary endpoint, intervention effect = testing order spirometry first, N_2_MBW second) was analyzed using the same model, with LCI_2.5_ as the outcome variable. LCI_2.5_ is defined as the number of lung turnovers required to reduce the alveolar N_2_ concentration to 1/40 (2.5) of its initial concentration. According to Perrem et al. [25], the following thresholds for LCI_2.5_ changes should be considered as clinically relevant in pwCF: clinically stable pwCF LCI variability > ±15% and for symptomatic pwCF LCI increase > 10% between follow‐ups. Correspondingly, changes in LCI_2.5_ of < ±15% are considered acceptable and independent of the preceding spirometry measurement. Accordingly, ±15% of the mean value of LCI_2.5_ is used to determine the equivalence range.

As with all rare diseases, the number of available patients was limited. For this reason, we did not include the LCI_2.5_ as an additional endpoint when estimating the number of necessary patients, as this would have required a larger number of cases. To calculate the number of cases for the primary endpoint needed to achieve 80% power with an *α* of 0.05 for our hypothesis with δ = 0.2, we used the formula of Chow et al. [26]. A more detailed description of the procedure can be found in Siyasinghe and Sooriyarachchi [27] and in the online supplement. This showed that a total of 44 patients were needed (22/group). If patients did not return or did not have two appointments without evidence of an exacerbation within the recruitment period, they were treated as loss to follow‐up.

All statistical analyses were performed using R version 4.2.1 and Microsoft Excel 2022 version 16.60.

## 3 Results

**Figure 1:**
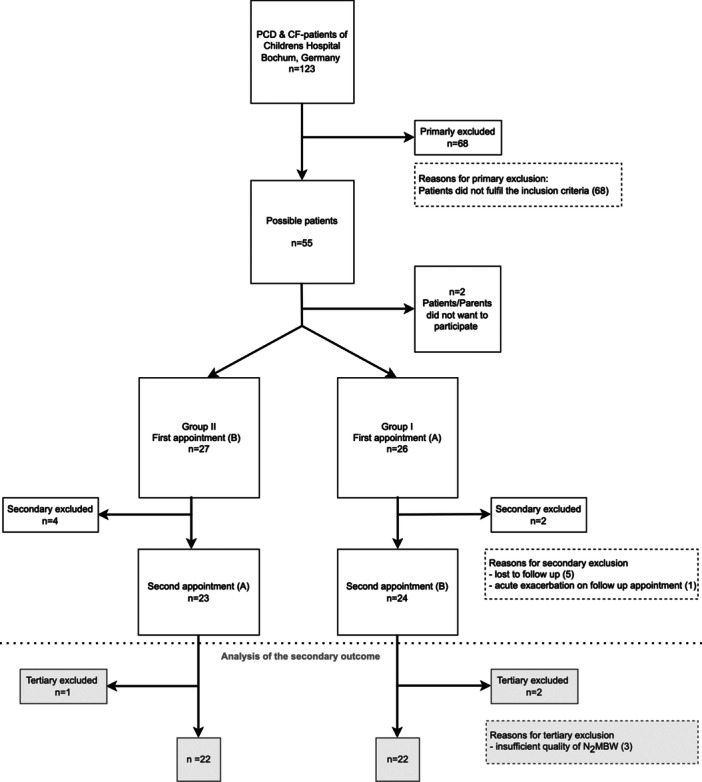
Flowchart of the recruitment process of the study. PCD = primary ciliary dyskinesia; CF = cystic fibrosis, N_2_MBW = nitrogen multiple breath washout, FEV_1_ = forced expiratory volume after 1 s, A: spirometry first, nitrogen multiple breath washout second, B: nitrogen multiple breath washout first, spirometry second.

In total, 53 subjects (pwCF *n* = 41, pwPCD *n* = 12, age range: 6–51 years (mean 17 ± 8.06 yrs)) were included. Six patients were excluded as five were lost to follow‐up and one had an acute exacerbation and was unable to return within the recruitment period. For the analysis of the influence of spirometry on LCI_2.5_, three further subjects were excluded because the quality of the N_2_MBW measurements did not meet the criteria of international guidelines [17] (pwCF *n* = 2, pwPCD *n* = 1, see Figure 1).

Clinical characteristics of the subjects are shown in Table 1. On average, there were 140 days (mean, median = 104 days, range: 22–350 days) between appointments.

**Table 1:** Clinical characteristics of the subjects.

		Group I	Group II	All
	*Appointment order, 1.→ 2*.	*A→ B*	*B→ A*	
All, *n*		24	23	47
Patients with PCD, *n*		9	2	11
Patients with CF, *n*		15	21	36
	*Ongoing modulator therapy, n (% of CF patients)*	7 (29%)	13 (57%)	20 (83%)
Age, years		16 ± 9.77	18 ± 5.88	17 ± 8.06
	Range	8–26	6–51	6–51
	6–12, *n*	8	6	14
	12–18, *n*	11	6	17
	> 18, *n*	5	11	16
	Median (interquartile range)	15 (7.5)	18 (9.5)	16 (10.5)
Female, *n* (%)		12 (50%)	9 (39%)	21 (39%)
BMI mean, kg/m^2^		20.49 ± 6.25	21.58 ± 3.81	21.02 ± 5.18
Ethnic	European, *n* (%)	15 (62.5%)	20 (87%)	35 (74.5%)
	Middle East, *n* (%)	9 (37.5%)	3 (13%)	12 (25.5%)
Stratified subgroups	*z*‐score FEV_1_ < (−1), *n* (%)	7 (29%)	7 (30%)	14 (30%)
	*z*‐score FEV_1_ > (−1), *n* (%)	17 (71%)	16 (70%)	33 (70%)
Bacterial colonization at inclusion, *n* (%)		16 (67%)	15 (65%)	31 (66%)

*Note:* Continuous variables are shown in mean ± standard deviation, categorical variables are shown in absolute values and percentages. *z*‐score: calculation according to Global Lung Initiative reference values (see Section 2.4). A = 1. spirometry, 2. nitrogen multiple breath washout; B = 1. nitrogen multiple breath washout, 2. spirometry.

Abbreviations: BMI = body mass index; CF = cystic fibrosis; FEV_1_ = forced expiratory volume after 1 s; PCD = primary ciliary dyskinesia.

Detailed information on bacterial colonization, genetics, and modulator application of pwCF and diagnostics for pwPCD is available in Supporting Information S1: Table E1–E4.

The results of the pulmonary function tests (PFTs) at both visits are shown in Table 2, showing a decrease in *z*‐score FEV_1_ between visits regardless of group randomization.

**Table 2:** Spirometry values in *z*‐scores and LCI_2.5_ measured in N_2_MBW.

		Group I	Group II	All
*z*‐score FEV_1_				
	First appointment	−0.36 ± 1.18	−0.12 ± 1.26	−0.25 ± 1.21
	Second appointment	−0.5 ± 1.17	−0.34 ± 1.17	−0.42 ± 1.16
*z*‐score FVC				
	First appointment	−0.1 ± 0.97	0.18 ± 1.01	0.04 ± 0.99
	Second appointment	−0.16 ± 1.11	0.09 ± 1.02	−0.04 ± 1.06
LCI_2.5_				
	First appointment	8.73 ± 1.81	8.95 ± 2.7	8.84 ± 2.28
	Second appointment	8.97 ± 1.76	8.8 ± 2.59	8.9 ± 2.19

*Note:* Values shown in mean ± standard deviation. Group I: sequence first appointment: 1. spirometry, 2. nitrogen multiple breath washout; second appointment vice versa. Group II: sequence first appointment: 1. nitrogen multiple breath washout, 2. spirometry; second appointment vice versa. *z*‐score: calculation according to Global Lung Initiative reference values (see Section 2.4).

Abbreviations: FEV_1_ = forced expiratory volume after 1 s; FVC = function vital capacity; LCI_2.5_ = lung clearance index; N_2_MBW = nitrogen multiple breath washout.

**Figure 2:**
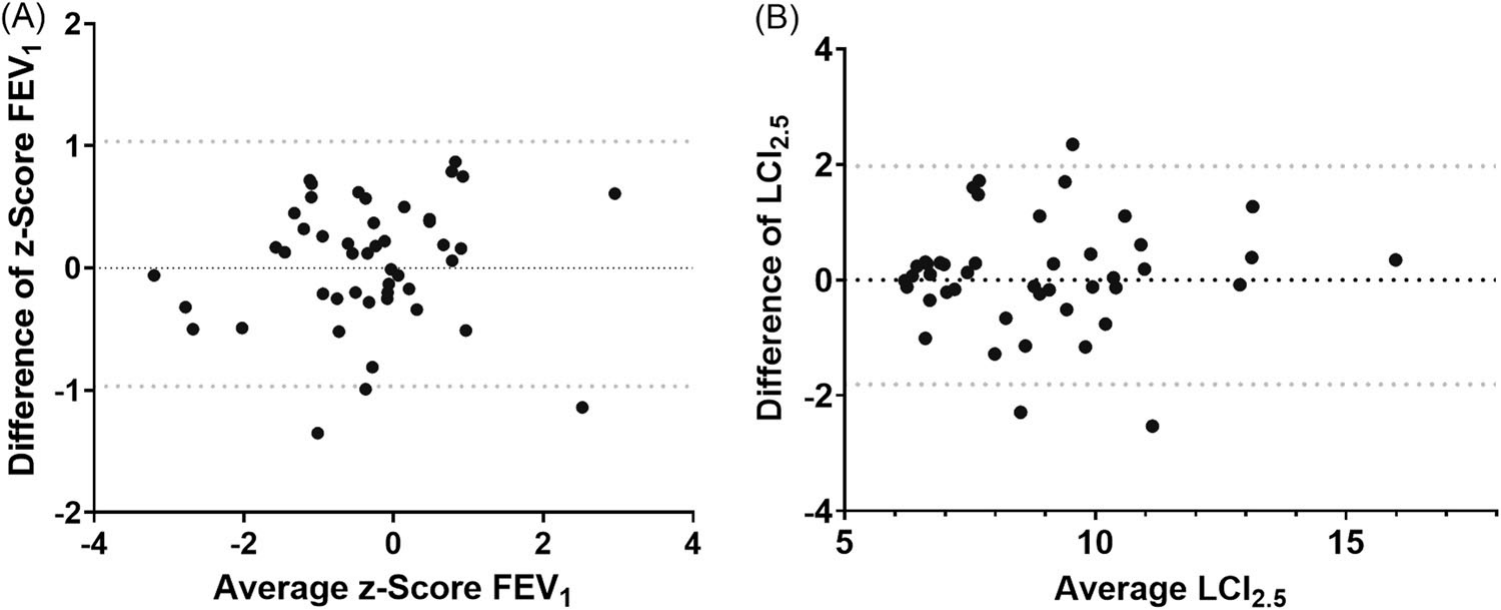
(A) Bland–Altman plot for *z*‐score FEV_1_ (*N* = 47); *x*‐axis: average of both appointments, *y*‐axis: difference between both measurements (Appointment A − Appointment B), gray dashed lines: upper and lower 95% confidence interval, black dots: mean values of the individuals in relation to the difference of the values. (B) Bland–Altman plot for LCI_2.5_ (*N* = 44); *x*‐axis: average of both appointments, *y*‐axis: difference between both measurements (Appointment A − Appointment B), gray dashed lines: upper and lower 95% confidence interval, black dots: mean values of the individuals in relation to the difference of the values, FEV_1_ = forced expiratory volume after 1 s, *z*‐score: calculation according to Global Lung Initiative reference values (see methods), LCI_2.5_ = lung clearance index. Appointment A: spirometry first, nitrogen multiple breath washout second. Appointment B: nitrogen multiple breath washout first, spirometry second.

The Bland–Altman plot visualized that the differences between the lung function tests at both visits of different order were not influenced by the lung function impairment as measured by *z*‐score FEV_1_ (Figure 2A) and LCI_2.5_ (Figure 2B). This means that regardless of baseline values, changes between visits are similar; lung function values in severely impaired patients do not vary more between visits than in less impaired patients.

The linear mixed model used to analyze the influence of N_2_MBW on spirometry (primary endpoint) showed a statistically significant period effect (*p* = 0.012, Table 3), meaning that the time between the two appointments influenced the second measurement, which was respected in the further analysis (for a detailed explanation, see Section Results, 4).

**Table 3:** Intervention‐ and period‐effects calculated by the linear mixed models.

		*z*‐score FEV_1_			LCI_2.5_	
			*p*			*p*
Intervention‐effect (95% confidence interval)	1. N_2_MBW	0.039	0.584*	1. Spirometry	−0.082	0.578*
	2. Spirometry	(−0.0765 to 0.1539)		2. N_2_MBW	(−0.3691 to 0.2054)	
Period‐effect		−0.177	0.012**		0.058	0.695

*Note:* Intervention‐ and period‐effects calculated by the linear mixed models; *z*‐score: calculation according to Global Lung Initiative reference values (see Section 2.4).

Abbreviations: FEV_1_ = forced expiratory volume after 1 s; LCI_2.5_ = lung clearance index; N_2_MBW = nitrogen multiple breath washout.

* *p* values for test of superiority

** *p* < 0.05.

**Figure 3:**
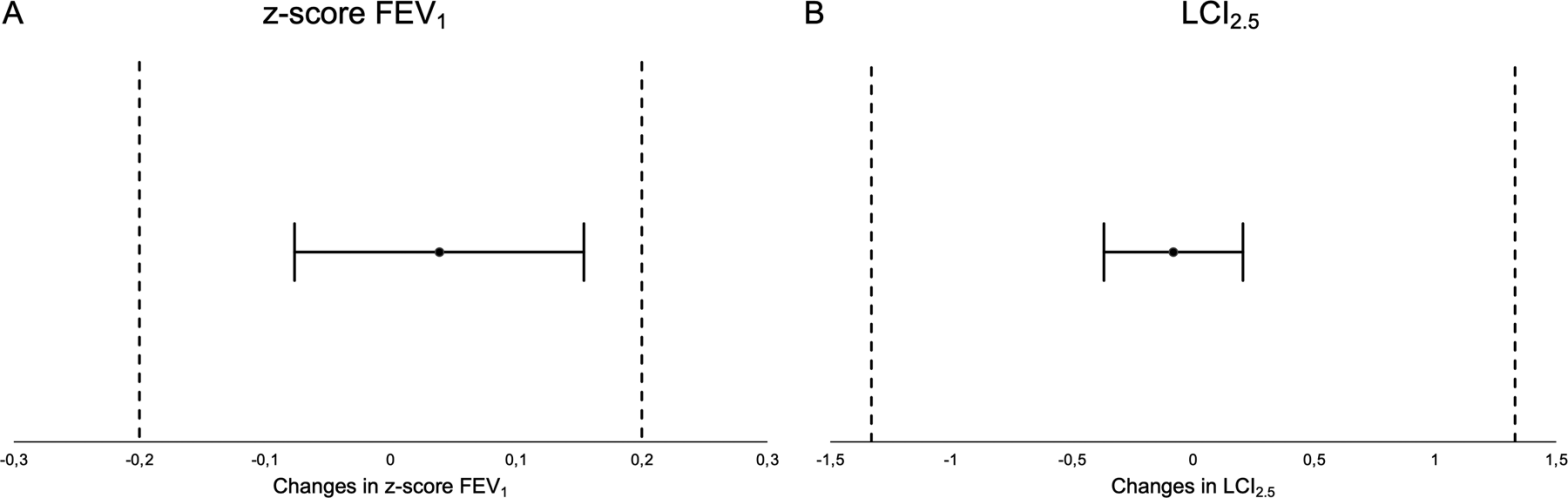
(A) Visualization of confidence interval of intervention effect on *z*‐score FEV_1_ with equivalence range; *x*‐axis: changes in *z*‐score FEV_1_; black line: 95% confidence interval of the Intervention effect N_2_MBW‐spirometry on *z*‐score FEV_1_; black dashed lines mark the equivalence range, black dot: intervention effect. (B) Visualization of confidence interval of intervention effect on LCI_2.5_; *x*‐axis: changes in LCI_2.5_; black line: 95% confidence interval of the intervention effect spirometry–N_2_MBW on LCI_2.5_, black dashed lines mark the maximum accepted change between appointments, black dot: intervention effect. FEV_1_ = forced expiratory volume after 1 s, *z*‐score: calculation according to Global Lung Initiative reference values (see Methods), N_2_MBW = nitrogen multiple breath washout; LCI_2.5_ = lung clearance index.

The estimates also showed a slightly positive intervention effect (N_2_MBW first, spirometry second) on *z*‐score FEV_1_ (Table 3), meaning that *z*‐score FEV_1_ in spirometry is slightly higher when N_2_MBW is performed first. To analyze whether this difference is within the equivalence range of ±0.2, the 90% confidence interval with a 95% level of error was estimated [28]. The confidence interval ranges from −0.0765 to 0.1539, completely within the equivalence range (Figure 3A). Thus, measuring N_2_MBW prior to spirometry may influence the results, but the difference is deemed to be negligible.

As a secondary outcome, the influence of spirometry on N_2_MBW was estimated using the same type of model, but with the LCI_2.5_ as the dependent variable.

The estimates showed a slightly negative intervention effect (spirometry first, N_2_MBW second) on LCI_2.5_ (Table 3), showing that LCI_2.5_ is slightly lower when spirometry is performed first. To interpret whether the changes are within the acceptable range, we calculated the 95% confidence interval which ranges from −0.3691 to 0.2054. The equivalence range is calculated as ±15% of the mean LCI value: The mean LCI_2.5_ is 8.87 units (Table 2), ±15% of this value are ±1.33 units, which is considerably greater than the width of the confidence interval (Figure 3B). With only 2.32% of the mean LCI_2.5_ value, the upper limit of the confidence interval is even lower than a 10% increase, which is the cutoff value for symptomatic patients. So again, there is a small effect, but it is within the expected range.

In contrast to the primary analysis, there is no statistically relevant period effect (*p* = 0.695, Table 3).

Individual changes between study appointments in *z*‐score FEV_1_ and LCI_2.5_ can be found in Supporting Information S2: Figure E1.

## 4 Discussion

### 4.1 Main Findings

Our study, using a randomized cross‐over design, shows that the *z*‐score FEV_1_ measured by spirometry in pwCF and pwPCD is not influenced by the immediately preceding N_2_MBW. In addition, this study corroborates that in reverse order LCI_2.5_ measured via N_2_MBW is not affected by spirometry. As expected, *z*‐score FEV_1_ decreased between the two study visits but independent of their order of N_2_MBW and spirometry.

### 4.2 Influencing Factors on N_2_MBW and Spirometry

Both spirometry and N_2_MBW, a common technique in children and adults [29], are standard tests for monitoring lung function in pwCF and pwPCD [11, 30, 31]. Routinely, N_2_MBW is performed before spirometry, to avoid possible changes in N_2_MBW results due to mobilized sputum caused by the forced expiratory maneuvers during spirometry [30]. But, there are concerns that N_2_MBW may influence spirometry: First, to measure the LCI_2.5_ by N_2_MBW, it is necessary to ventilate with 100% oxygen for several minutes. It has been hypothesized that the use of 100% oxygen to measure the LCI in infants could alter the breathing pattern, hence affecting LCI results [32]. More recent studies with larger cohorts and older patients could not replicate this hypothesis [33]. It is conceivable, but not yet directly investigated, that this oxygen exposure may affect spirometry values. Indeed, in healthy children under general anesthesia ventilated with high inspired oxygen fractions, a decrease in FRC measured by N_2_MBW has been reported up to 2 h after extubation [33]. In addition, it has been suggested that hyperoxia may lead to increased ventilation heterogeneities [33, 34], but there is no significant evidence from clinical trials [34, 35]. However, there are no studies with pwCF or pwPCD in children, so the influence of the possible hyperoxaemia in N_2_MBW on spirometry values in general cannot be excluded. Furthermore, both N_2_MBW and spirometry depend on patient cooperation: for spirometry, permanent motivation and guiding, as well as active participation, are required to perform spirometry correctly [16]. N_2_MBW requires a stable tidal breathing pattern, which may be difficult to achieve in young children without appropriate distraction like watching a video and needs at least two correct measurements for valid interpretation [17, 36]. This is time‐consuming especially in more advanced disease stages, which can lead to reduced patient motivation for further testing [36], such as spirometry and some may even refuse it altogether. In our clinical experience, flexibility in the sequence of PFTs is important to avoid long waiting periods and reduce the length of regular follow‐up visits to improve patients' compliance. Despite all these possible factors influencing the spirometry results, the usual order is still followed, although there is no guideline recommendation for this order.

### 4.3 Comparison With Previous Studies

Two studies have investigated the influence of spirometry on MBW, both using a test–retest design: in 2015, Subbarao et al. [15] were the first to perform MBW immediately before and after forced expiratory maneuvers in sedated infants (4–30 months) and reported a significant decrease in LCI after performing RVRTC in infants with respiratory disease compared to healthy infants. However, as usual for this age group, the MBW was performed with a sulfur hexafluoride (SF_6_)/helium gas mixture. The results of an MBW test with a tracer gas such as SF_6_ are not interchangeable with those of N_2_ and 100% oxygen [29, 37]. In addition, RVRTC is used to measure FEV_1_ in infants instead of spirometry. Although RVRTC is a safe technique, it requires higher driving pressures than spirometry and the methods for measuring FEV_1_ are different [38]. In conclusion, the results of this study cannot be applied to older subjects performing MBW with N_2_ and 100% oxygen and spirometry. In 2018, Au et al. [14] addressed this issue in pwCF (aged 6–18 years) by performing N_2_MBW before and immediately after spirometry and found that spirometry had no short‐term effect on N_2_MBW. However, the design of the study did not allow for randomization, nor did it allow the influence of N_2_MBW on spirometry to be investigated. Nonetheless, their results are consistent with our findings. As the mean change in LCI_2.5_ in our cohort is considerably lower than the variability in LCI_2.5_ recently described by Perrem et al. [25] as clinically relevant, we can conclude that LCI_2.5_ was not influenced by previous spirometry performance.

To the best of our knowledge, no other study has investigated the influence of N_2_MBW on spirometry values. This study is the first to demonstrate that N_2_MBW has no effect on spirometry and in a randomized cross‐over design, further confirms that spirometry has no influence on N_2_MBW measurement.

### 4.4 Period Effect

There is a statistically significant period effect of −0.177 (*p* = 0.012) for *z*‐score FEV_1_ meaning that the time interval between the two appointments had an influence on the second measurement. However, this does not alter the significance of our results. This can be explained in two ways: First, we used a linear mixed model to analyze our results to account for the period effect while estimating the intervention effect. Thus, the main point of the analysis remained unaffected by the potential decline of the variables with time in the individuals. Second, a decrease in *z*‐score FEV_1_ is in line with expectations because CF and PCD are characterized by a progressive decline of lung function over time [4, 39, 40]. In addition, by using patients from our clinic to determine the equivalence range, we are able to directly assess the period effect: it is within the equivalence range of ±0.2 which is not greater than expected for this cohort. In conclusion, a significant period effect is not uncommon in this cohort and has been accounted in the analysis by implementation in the linear mixed model.

### 4.5 Strengths and Limitations

The design of a prospective, randomized cross‐over study is the major strength of this study. The inclusion of the study in the regular outpatient control visits is one of the limitations. This resulted in a long interval between appointments, with the constant risk of exacerbations and disease progression and led to the significant period effect. In eight subjects, the follow‐up visit had to be postponed to the next or even later regular follow‐up visit because of an acute exacerbation. Two measurements on the same day or an interval of a few days between two study visits may have reduced these problems but it might also have led to a decrease in willingness to participate, risking that fewer subjects would be enrolled. However, because of the randomization, it is unlikely that one group had to postpone more visits due to exacerbations than the other.

It is irritating that only the primary outcome showed a significant period effect, especially as N_2_MBW is a more sensitive method than spirometry [11]. However, there were no outliers in the measurements that could have influenced the period effect of the secondary outcome.

Our study cohort is very heterogeneous due to the inclusion of pwCF and pwPCD and the wide age range. It would have been more appropriate to conduct two studies for each disease, but this was difficult due to the small number of patients. Therefore, we used Bland–Altman plots to show that changes in *z*‐score FEV_1_ and LCI_2.5_ did not depend on the average value representing the baseline values, which were highly variable within the cohort, so heterogeneity did not seem to affect the results.

Although the study was powered only for the primary endpoint (influence of N_2_MBW on *z*‐score FEV_1_), we were still able to show non‐inferiority for the secondary endpoint. Because there is no reason to suspect that the estimate is biased, the conclusion that not only N_2_MBW does not influence the *z*‐score FEV_1_ but also that spirometry does not influence LCI_2.5_ is valid. This study is the first to demonstrate the independence of the order of N_2_MBW and spirometry in pwCF and pwPCD in a randomized cross‐over design. However, as discussed above, the challenging conditions encountered when studying in real life lead to limitations why further studies are needed to evaluate our findings in larger cohorts and in other patient groups.

## 5 Conclusion

In our cohort of pwPCD and pwCF, lung function measurements N_2_MBW and spirometry do not influence each other. Our results suggest that a greater flexibility in practice is possible, as there is no need to adhere to a prescribed order without the risk of falsifying results.

## Author Contributions

**Anna Charlotte Schoop:** writing – original draft, visualization, conceptualization, data curation, investigation, writing – review and editing, methodology. **Robin Denz:** formal analysis, visualization, writing – review and editing, conceptualization. **Christoph Maier:** conceptualization, writing – original draft, writing – review and editing, methodology. **Folke Brinkmann:** writing – review and editing, conceptualization, methodology, investigation, data curation. **Thomas Lücke:** supervision, writing – review and editing. **Anne Schlegtendal:** data curation, conceptualization, writing – original draft, writing – review and editing, methodology, investigation, project administration. All authors read and approved the published version of the manuscript.

## Ethics Statement

The study was approved by the Ethics Committee of the Ruhr‐University of Bochum (No. 21. 7210) on 03.28.2021.

## Conflicts of Interest

The authors declare no conflicts of interest.

## Supporting information

E‐tables 13.

Figure E1.

Image legend E‐Figure 13.

Sample Size Calculation Appendix.

## Data Availability

The data that support the findings of this study are available from the corresponding author upon reasonable request.
