# Supplementary material for: Randomized Cross‐Over Analysis of the Influence of Nitrogen Multiple Breath Washout on Spirometry in Monitoring Lung Function in Patients With Cystic Fibrosis and Primary Ciliary Dyskinesia
Source: Pediatr Pulmonol. 2025 Jul 10;60(7):e71189. doi: 10.1002/ppul.71189 (PMC12243717; doi:10.1002/ppul.71189)
Supplement: Supplementary file 1 — E‐tables 13. [file PPUL-60-0-s004.docx]

| **E1**. Bacterial colonisation | | | | | | |
| --- | --- | --- | --- | --- | --- | --- |
|  | all | | group I | | group II | |
|  | PCD | CF | PCD | CF | PCD | CF |
| Number of subjects (n) | 11 | 36 | 9 | 15 | 2 | 21 |
| Pathological bacterial colonization | 5 (45.45%) | 26 (72.22%) | 5 (55.56%) | 11 (73.33%) | 0 (0%) | 15 (71.43%) |
| Acinetobacter junii | 0 (0%) | 1 (2.78%) | 0 (0%) | 0 (0%) | 0 (0%) | 1 (4.76%) |
| Aspergillus fumigatus | 0 (0%) | 1 (2.78%) | 0 (0%) | 0 (0%) | 0 (0%) | 1 (4.76%) |
| Citrobacter koseri | 0 (0%) | 1 (2.78%) | 0 (0%) | 1 (6.67%) | 0 (0%) | 0 (0%) |
| Escherichia coli | 1 (9.09%) | 1 (2.78%) | 1 (11.11%) | 1 (6.67%) | 0 (0%) | 0 (0%) |
| Haemophilus influenzae | 0 (0%) | 1 (2.78%) | 0 (0%) | 1 (6.67%) | 0 (0%) | 0 (0%) |
| Moraxella catarrhalis | 1 (9.09%) | 0 (0%) | 1 (11.11%) | 0 (0%) | 0 (0%) | 0 (0%) |
| Proteus mirabilis | 0 (0%) | 2 (5.56%) | 0 (0%) | 1 (6.67%) | 0 (0%) | 1 (4.76%) |
| Pseudomonas aeruginosa | 1 (9.09%) | 4 (11.11%) | 1 (11.11%) | 1 (6.67%) | 0 (0%) | 3 (14.29%) |
| Serratia liquefaciens | 0 (0%) | 1 (2.78%) | 0 (0%) | 0 (0%) | 0 (0%) | 1 (4.76%) |
| Serratia marcescens | 2 (18.18%) | 0 (0%) | 2 (22.22%) | 0 (0%) | 0 (0%) | 0 (0%) |
| Staphylococcus aureus | 0 (0%) | 14 (38.89%) | 0 (0%) | 6 (40%) | 0 (0%) | 8 (38.1%) |
| Values shown in absolute values and percentages.  PCD = Primary Ciliary Dyskinesia  CF = Cystic Fibrosis  Group I = 1. Appointment: spirometry 🡪 Nitrogen Multiple Breath Washout (N_2_MBW). 2. Appointment: N_2_MBW 🡪 spirometry  Group II = 1. Appointment: N_2_MBW 🡪 spirometry. 2. Appointment: spirometry 🡪 N_2_MBW | | | | | | |

| **E2**. CFTR-Therapy for CF-Patients | | | |
| --- | --- | --- | --- |
|  | all | group I | group II |
| Number of patients | 36 | 15 | 21 |
| CFTR-Modulators | 20 (55.56%) | 7 (46.67%) | 13 (61.9%) |
| Ivacaftor | 0 | 0 | 0 |
| Lumacaftor/Ivacaftor | 2 | 0 | 2 |
| Tezacaftor/Ivacaftor | 0 | 0 | 0 |
| Tezacaftor/Ivacaftor/Elexacaftor | 3 | 1 | 2 |
| Tezacaftor/Ivacaftor/Elexacaftor + Ivacaftor evenings | 14 | 5 | 9 |
| Values shown in absolute values and percentages.  CF = Cystic Fibrosis  CFTR = cystic fibrosis transmembrane conductance regulator  Group I = 1. Appointment: spirometry 🡪 Nitrogen Multiple Breath Washout (N_2_MBW). 2. Appointment: N_2_MBW 🡪 spirometry  Group II = 1. Appointment: N_2_MBW 🡪 spirometry. 2. Appointment: spirometry 🡪 N_2_MBW | | | |

| **E3**. Genetic of CF-Patients | | | |
| --- | --- | --- | --- |
|  | all | group I | group II |
| Number of subjects (n) | 36 | 15 | 21 |
| del F508 homozygot | 15 (41.7%) | 4 (26.7%) | 11 (52.4%) |
| del F508 heterozygot | 16 (44.4%) | 8 (53.3%) | 8 (38.1%) |
| others | 5 (13.9%) | 3 (20%) | 2 (9.5%) |
| Values shown in absolute values and percentages.  CF = Cystic Fibrosis  Group I = 1. Appointment: spirometry 🡪 Nitrogen Multiple Breath Washout (N_2_MBW). 2. Appointment: N_2_MBW 🡪 spirometry  Group II = 1. Appointment: N_2_MBW 🡪 spirometry. 2. Appointment: spirometry 🡪 N_2_MBW | | | |

| **E4.** Diagnostic of PCD-Patients | | | | |
| --- | --- | --- | --- | --- |
|  |  | all | group I | group II |
| Number of subjects (n) | | 11 | 9 | 2 |
| Situs |  |  |  |  |
|  | Situs solitus | 9 | 8 | 1 |
|  | Situs inversus | 2 | 1 | 1 |
| HVMA |  |  |  |  |
|  | Immotile | 6 | 4 | 2 |
|  | Dyskinetic | 4 | 4 | 0 |
|  | No information | 1 | 1 | 0 |
| TEM |  |  |  |  |
|  | IDA defects + Microtubule disorganization | 1 | 1 | 0 |
|  | IDA + ODA defect | 4 | 3 | 1 |
|  | Normal | 3 | 2 | 1 |
|  | No information | 3 | 3 | 0 |
| IF |  |  |  |  |
|  | Normal | 3 | 3 | 0 |
|  | DNAH5 | 6 | 4 | 2 |
|  | No information | 2 | 2 | 0 |
| Genetic |  |  |  |  |
|  | CCDC39 | 1 | 1 | 0 |
|  | DNAH11 | 2 | 2 | 0 |
|  | DNAI1 | 1 | 1 | 0 |
|  | DNAI2 | 1 | 1 | 0 |
|  | RSPH4A | 1 | 1 | 0 |
|  | SPAG1 | 1 | 1 | 0 |
|  | DNAH5 | 4 | 2 | 2 |
| Values shown in absolutes | | | | |
| PCD = Primary Ciliary Dyskinesia | | | | |
| Group I = 1. Appointment: spirometry 🡪 Nitrogen Multiple Breath Washout (N_2_MBW). 2. Appointment: N_2_MBW 🡪 spirometry | | | | |
| Group II = 1. Appointment: N_2_MBW 🡪 spirometry. 2. Appointment: spirometry 🡪 N_2_MBW | | | | |
| HVMA = High-frequence video microscopy analysis | | | | |
| TEM = Transmission electron microscopy | | | | |
| IF = Immunofluorescens analysis | | | | |
