# Supplementary figures and images for: Randomized Cross‐Over Analysis of the Influence of Nitrogen Multiple Breath Washout on Spirometry in Monitoring Lung Function in Patients With Cystic Fibrosis and Primary Ciliary Dyskinesia
Source: Pediatr Pulmonol. 2025 Jul 10;60(7):e71189. doi: 10.1002/ppul.71189 (PMC12243717; doi:10.1002/ppul.71189)

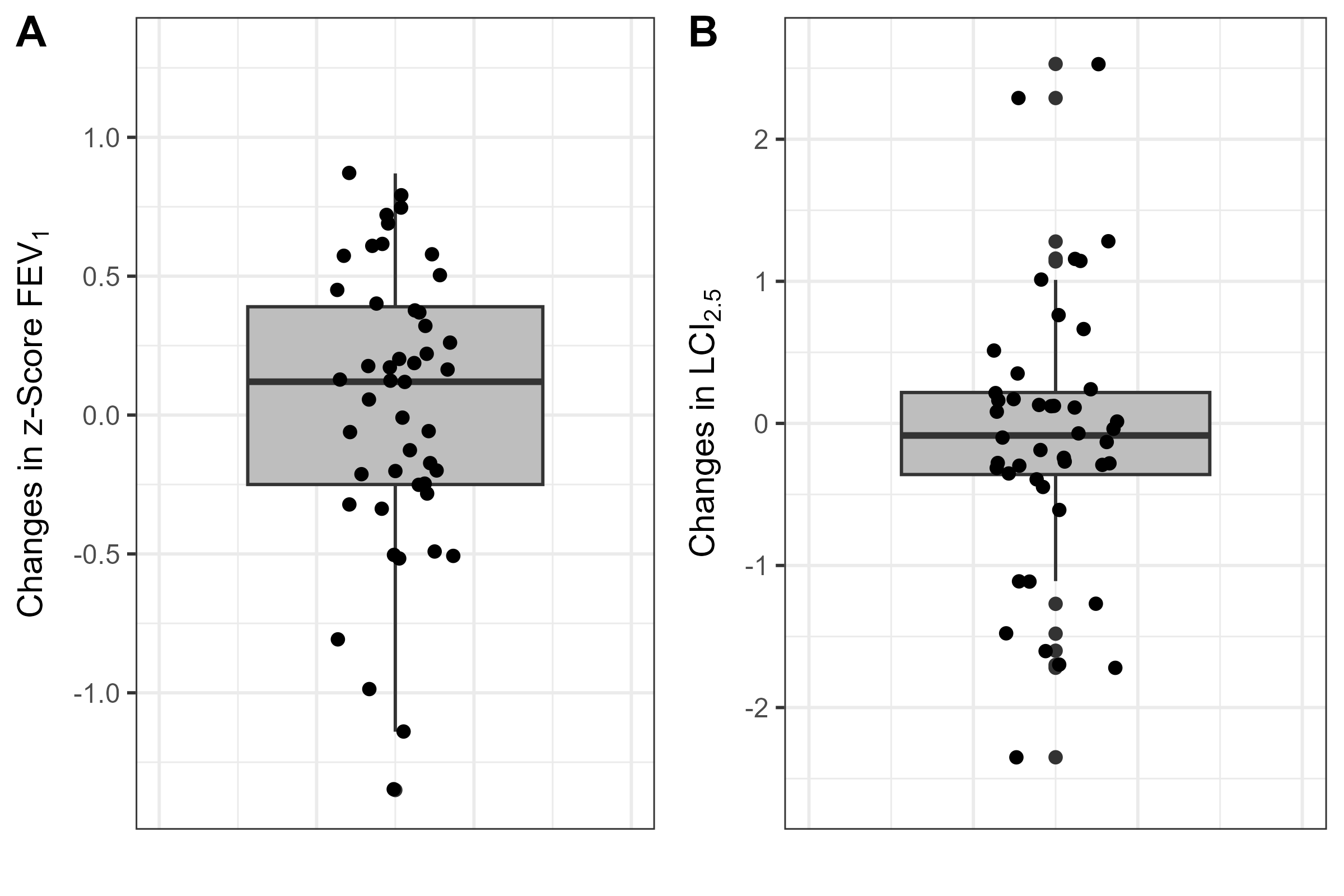

Supplement: Supplementary file 2 — Figure E1. [file PPUL-60-0-s001.tif]
