# Supplementary material for: Randomized Cross‐Over Analysis of the Influence of Nitrogen Multiple Breath Washout on Spirometry in Monitoring Lung Function in Patients With Cystic Fibrosis and Primary Ciliary Dyskinesia
Source: Pediatr Pulmonol. 2025 Jul 10;60(7):e71189. doi: 10.1002/ppul.71189 (PMC12243717; doi:10.1002/ppul.71189)
Supplement: Supplementary file 3 — Image legend E‐Figure 13. [file PPUL-60-0-s002.docx]

Figure E1

(A) Boxplot of the difference in z-score FEV_1_ (N=47); y-axis: changes in z-score FEV_1_; each point additionally represents one individuum; horizontal black line: median; grey box: Interquartile range, vertical black lines: Whiskers

(B) Boxplot of the difference in LCI_2.5_ (N=44); y-axis: changes in LCI_2.5_  ; each point additionally represents one individuum; horizontal black line: median; grey box: Interquartile range, vertical black lines: Whiskers

FEV_1_ = forced expiratory volume after 1 second, z-score: calculation according to Global Lung initiative reference values (see methods); LCI_2.5_ = lung clearance index
