# Supplementary material for: Randomized Cross‐Over Analysis of the Influence of Nitrogen Multiple Breath Washout on Spirometry in Monitoring Lung Function in Patients With Cystic Fibrosis and Primary Ciliary Dyskinesia
Source: Pediatr Pulmonol. 2025 Jul 10;60(7):e71189. doi: 10.1002/ppul.71189 (PMC12243717; doi:10.1002/ppul.71189)
Supplement: Supplementary file 4 — Sample Size Calculation Appendix. [file PPUL-60-0-s003.docx]

**Sample Size Calculation:**

Below we give more details on the sample size calculation that was performed before the study. The following equivalence hypothesis were used:

$$H_{0}=|\mu_{1}-\mu_{0}| \geq\delta$$

$$H_{1}=\left| \mu_{1}-\mu_{0} \right|<\delta$$

In these hypothesis, $\mu_{0}$ is defined as the mean of the FEV_1_ z-score in the control group (spirometry first, then MBW) and $\mu_{1}$ as the mean of the FEV_1_ z-score in the experimental group (MBW first, then spirometry). Additionally, $\delta$ is defined as the equivalence margin.

The study is a randomized crossover design. We therefore used the method given in Chow et al. (2003) for the sample size calculation. An in-depth explanation of the method is also given in Siyasinghe et al. (2011). The following parameters were used for the concrete calculation:

- α = 0.05 (significance level)
- Statistical Power = 0.8
- $\mu_{1}- \mu_{0}=0$
- $\sigma_{BR}^{2}=1.1368$
- $\sigma_{BT}^{2}=1.2407$
- $\sigma_{WR}^{2}=0.1019$
- $\sigma_{WT}^{2}=0.1154$
- $\rho=0.8$
- $\delta=0.2$

Here, $\sigma_{BR}^{2}$ is the variance between patients in the control group and $\sigma_{BT}^{2}$ is the variance between patients in the experimental group. These values were estimated using data from 17 unrelated patients from the same clinic. The mean difference is set to 0 because we expect no true effect of the treatment group, as is standard in non-inferiority and equivalence trials. $\sigma_{WR}^{2}$ and $\sigma_{WT}^{2}$ are the variances between measurements at different points in time of the same patient in the control and experimental group respectively. The amount of this variation was estimated to be on average about 16% by Svedberg et al. (2018), which when applied to the means in the control group, estimated from the 17 unrelated patients as before, results in the shown values. $\rho$ was assumed to be high and therefore set to 0.8.

According to Siyasinghe et al. (2011) the full variance for the sample size calculation was then estimated using the equation:

$$\sigma_{m}^{2}= \sigma_{BT}^{2}+ \sigma_{BR}^{2}-2\rho\sigma_{BT}\sigma_{BR}+\sigma_{WT}^{2}+\sigma_{WR}^{2},$$

which resulted in a final $\sigma_{m}^{2}$ of 0.69464. The sample size calculation was then performed using the TrialSize R package. The following R code can be used to replicate the sample size calculation using R version 4.3.3:

library(TrialSize)

n_per_group <- TwoSampleCrossOver.Equivalence(0.05, 0.8, 0.2, 0.69464, 0)

ceiling(n_per_group) * 2

**Literature:**

Chow, S.-C.; Shao, J. & Wang, H. (2003) Sample Size Calculations in Clinical Research. Marcel Dekker, Inc.

Siyasinghe, N. M. & Sooriyarachchi, M. R. (2011) Guidelines for Calculating Sample Size in 2x2 Crossover Trials: A Simulation Study. Journal of the National Science Foundation of Sri Lanka, 39, pp. 77-89

Svedberg, M.; Gustafsson, P. M.; Robinson, P. D.; Rosberg, M. & Lindblad, A. (2018) Variability of Lung Clearance Index in Clinically Stable Cystic Fibrosis Lung Disease in School Age Children. Journal of Cystic Fibrosis, 17, pp. 236-241
